# Supplementary material for: Graft-infiltrating host dendritic cells play a key role in organ transplant rejection
Source: Nat Commun. 2016 Aug 24;7:12623. doi: 10.1038/ncomms12623 (PMC4999515; doi:10.1038/ncomms12623)
Supplement: Supplementary Information — Supplementary Figures 1-4 [file ncomms12623-s1.pdf]

SUPPLEMENTARY FIGURES

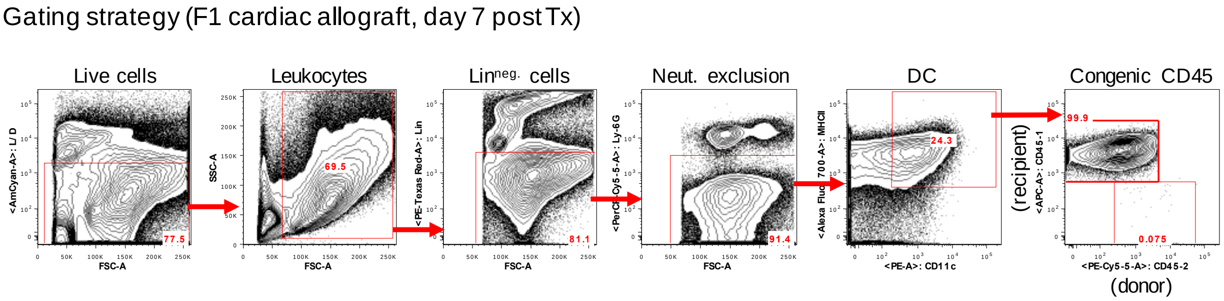

Supplementary Figure 1:

Full gating strategy of donor and recipient graft leukocytes. Leukocytes were isolated from a CD45.2 F1 (BALB/c x B6) allograft transplanted to CD45.1 B6 recipient on day 7 after transplantation and analyzed by flow cytometry. A representative sample is shown. Depicted is the full gating strategy resulting in the flow plots at the time points shown in Fig. 1a.

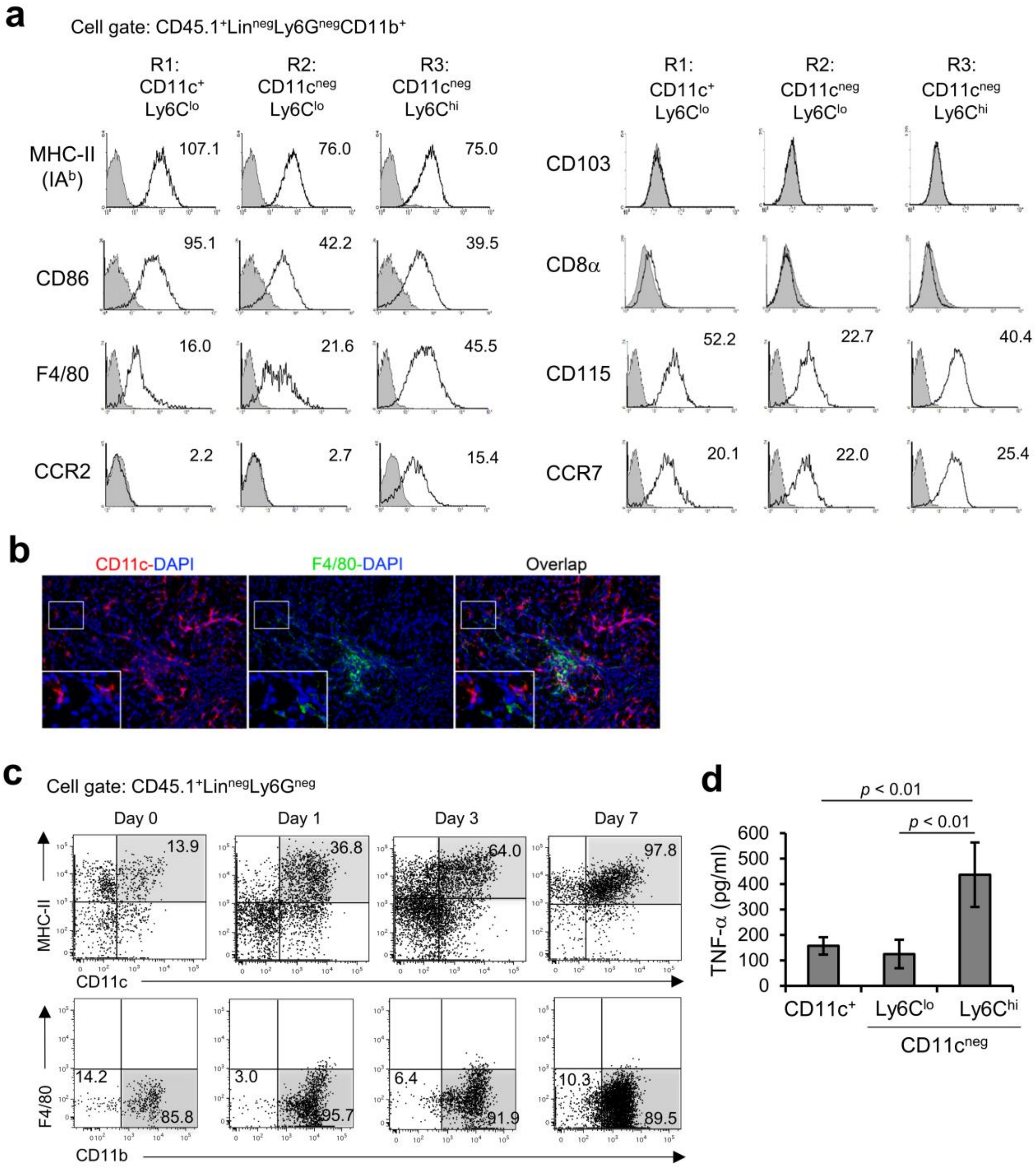

Supplementary Figure 2. *See legend on next page.*

Supplementary Figure 2 *(see image on previous page).*

Characterization of recipient-derived $CD11c^{+}$ and $CD11c^{neg}$ myeloid cell subsets in heart allografts. **(a)** Cells isolated on day 7 from CD45.2 BALB/c allografts transplanted to CD45.1 B6 recipients in Fig. 1c were analyzed by flow cytometry. After gating on $CD45.1^{+}Lin^{neg}Ly6G^{neg}CD11b^{+}$ cells, $CD11c^{+}$ and $CD11c^{neg}$ subsets (R1, R2, & R3 as defined in Fig. 1c) were analyzed for the surface markers shown. Representative histograms are shown. **(b)** Representative micrographs of heart allograft tissue stained for CD11c (red) and macrophage marker F4/80 (green). Cell nuclei were stained with DAPI (blue). Overlay demonstrates that the vast majority of $CD11c^{+}$ cells are $F4/80^{neg}$ while most $F4/80^{+}$ cells are $CD11c^{neg}$. Magnification 200x. **(c)** Leukocytes extracted from heart allografts in Fig. 1a were analyzed by flow cytometry to further establish the phenotype of recipient DCs. After gating on recipient myeloid cells ($CD45.1^{+}Lin^{neg}Ly6G^{neg}$), $CD11c^{+}MHC\text{-}II^{+}$ (DCs) were gated (top panels, shaded right upper quadrant) and analyzed for F4/80 and CD11b expression. The vast majority were $F4/80^{neg}$ and $CD11b^{+}$ (lower panels, shaded right lower quadrant). **(d)** TNF-α production by sorted $CD11c^{+}$ and $CD11c^{neg}$ subsets was quantified by ELISA as described for IL-12p70 (Fig. 1d). *P* values were generated by 1-way ANOVA followed by Tukey-Kramer multiple comparison test.

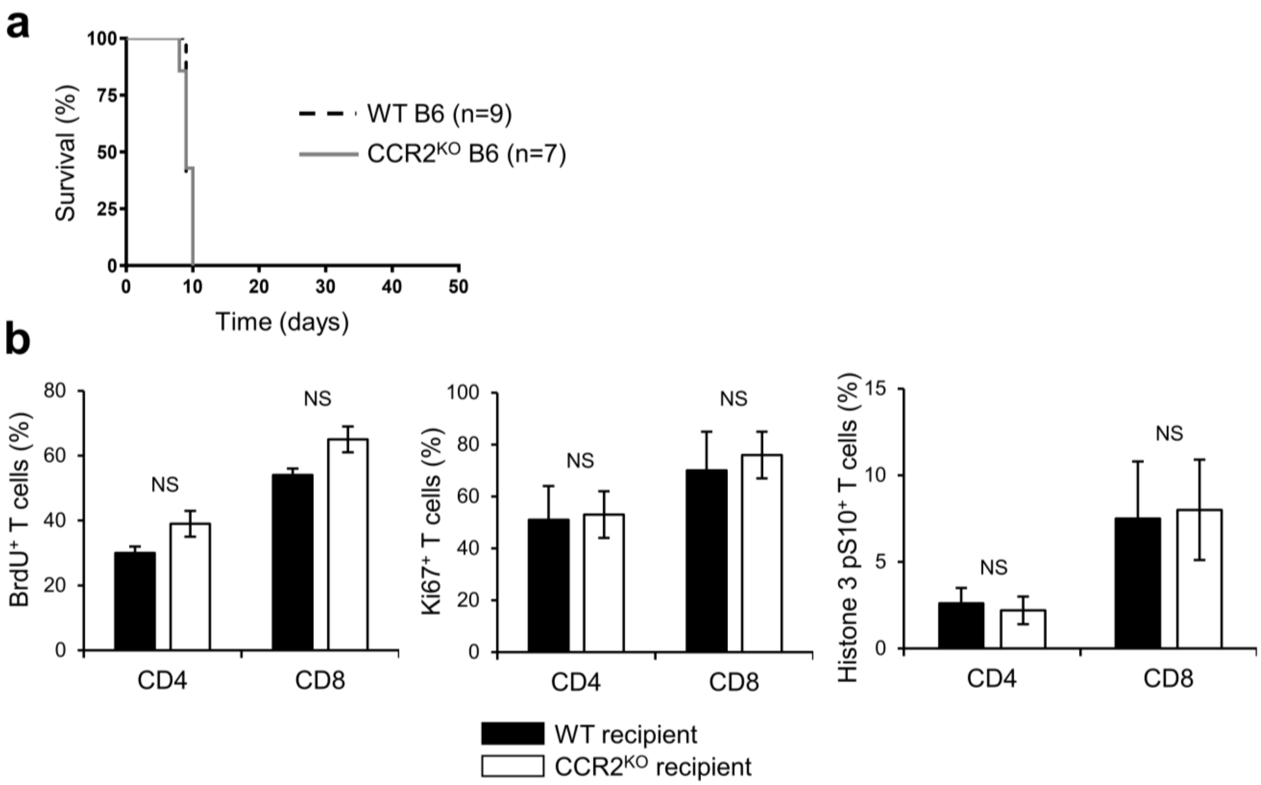

Supplementary Figure 3.

Allograft survival and intragraft T cell proliferation are not influenced by CCR2 deficiency in the recipient. (a) BALB/c heart allografts were transplanted to either WT or CCR2^{KO} B6 mice and graft survival was monitored by daily palpation of heart contractions. Kaplan-Meier graft survival plot is shown. **(b)** Heart transplantation was performed as in (a) except that all grafts were harvested on day 7 after transplantation and analyzed for T cell proliferation by flow cytometry (BrdU uptake) and by immunostaining (Ki67 and Histone 3 pS10). Bars are mean ± SD. N = 4 mice/group. Statistical analysis was done with Student's *t* test for unpaired samples.

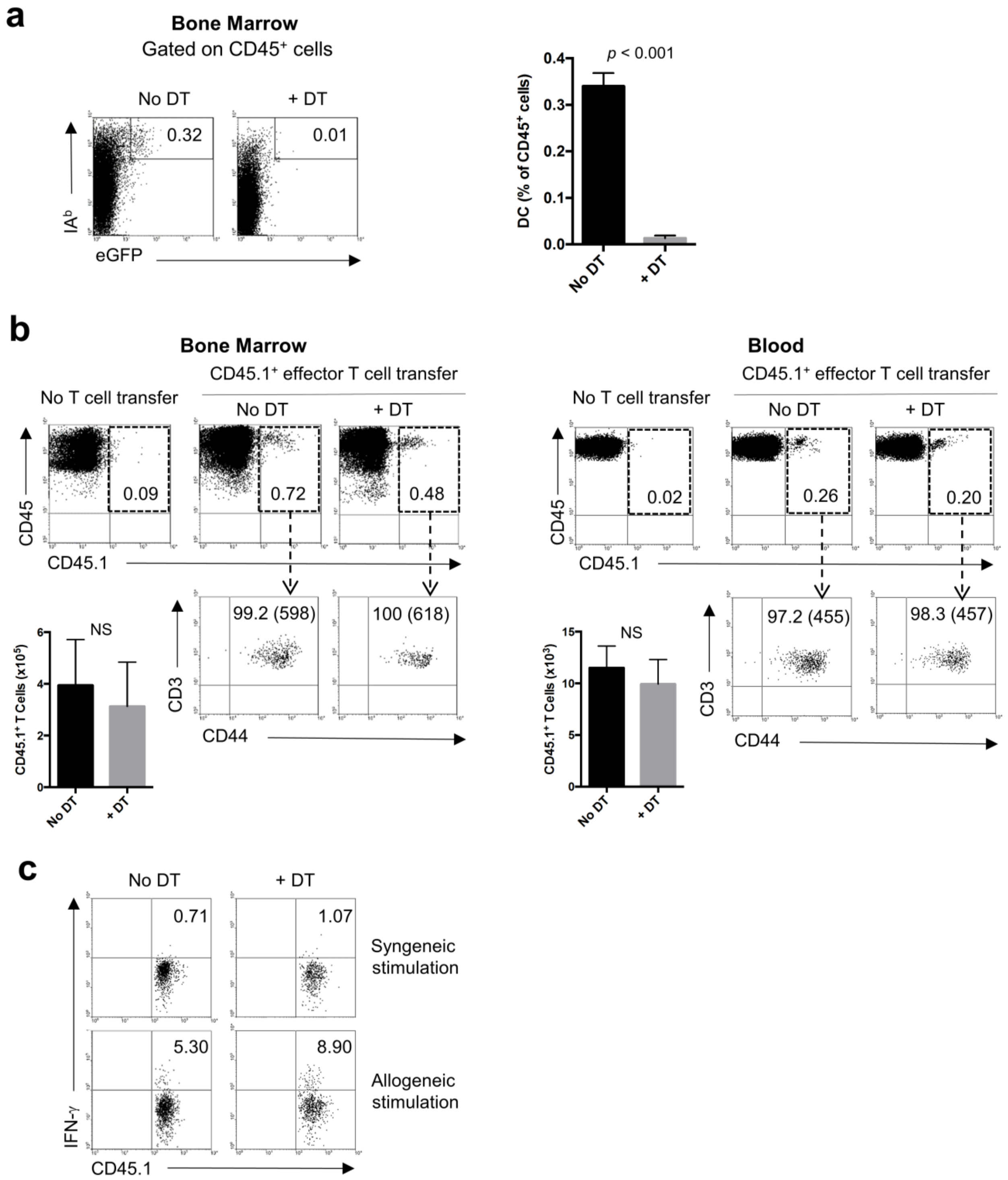

Supplementary Figure 4. *See legend on next page.*

Supplementary Figure 4 *(see image on previous page).*

Engraftment of adoptively transferred effector T cells is not influenced by DC depletion of host. Effector /memory ($CD44^{hi}$) T cells were sorted from B6 (CD45.1) mice primed with BALB/c splenocytes and transferred i.v. (8×10^6 T cells/mouse) to splenectomized CD11c-eGFP-DTR → $LT\beta R^{KO}$ B6 bone marrow chimeras (CD45.2). Mice were injected i.p. with DT every other day starting 1 day before T cell transfer (+ DT; n = 3). Control chimeras (No DT) received the same number of effector/memory T cells but no DT (n = 2). On day 21, bone marrow and blood were analyzed by flow cytometry. Representative flow plots and bar graphs (Mean ± SD) are shown. **(a)** Depletion of DCs in bone marrow of DT-treated mice. All $CD45^+IA^{b+}eGFP^+$ cells were CD11c+ (not shown). **(b)** Detection of transferred ($CD45.1^+$) effector ($CD3^+CD44^{hi}$) T cells in bone marrow and blood of DT-treated and untreated mice. Flow plots from mice that received neither effector T cells nor DT are shown as negative controls. Numbers in parentheses are MFI of CD44 expression. NS: not significant. **(c)** IFN-γ production by transferred ($CD45.1^+$) effector T cells was determined by flow cytometry after incubating bone marrow cells with either allogeneic (BALB/c) or syngeneic (B6) splenocytes *ex vivo* for 16 hrs in the presence of Golgi plug. In (a and b) *p* values were calculated with Student's *t* test for unpaired samples.
